# *In vitro *study of the effect of diesterified alkoxyglycerols with conjugated linoleic acid on adipocyte inflammatory mediators

**DOI:** 10.1186/1476-511X-9-36

**Published:** 2010-04-06

**Authors:** Aurelio Ocaña, Cristina Gómez-Asensio, Elena Arranz-Gutiérrez, Carlos Torres, Francisco Javier Señoráns, Guillermo Reglero

**Affiliations:** 1 Departamento de Química - Física Aplicada, Sección de Ciencias de la Alimentación, Facultad de Ciencias, Universidad Autónoma de Madrid, C/Fco, Tomás y Valiente 7, Madrid E 28049, Spain

## Abstract

**Background:**

Adipocytes contribute to inflammation and the innate immune response through expression of inflammatory mediators. High levels of these mediators have been related to chronic inflammation state and insulin resistance, cardiovascular diseases and diabetes type 2, among other disorders. 3-octadecylglycerol (batyl alcohol) has been described as an inflammatory agent, whereas Conjugated Linoleic Acid (CLA) is considered effective against obesity. In this study we examined the anti-inflammatory activity and mechanisms of modified alkoxyglycerols. Tumor necrosis factor (TNF-α) activated mature adipocytes were used as cellular model of inflammation. Secreted levels and gene expressions of some inflammatory mediators, such as the adipokines, interleukin (IL)-1β, IL-6 and IL-10; and the levels of leptin and adiponectin hormones were quantified in presence and absence of alkoxyglycerols and when human adipocyte cells were or not activated by TNF-α. The aim of this study is to describe the effects of nonesterified alkoxyglycerols, CLA and diesterified alkoxyglycerols with CLA (DEA-CLA) and check if they present beneficial properties using an in vitro model of some chronic diseases related to the inflammatory process, such as obesity, using human mature adipocytes activated with TNF-α.

**Results:**

Our data suggest that DEA-CLA, product of the esterification between the CLA and batyl alcohol, present beneficial effects on adipocytes close to observed and described for CLA (i.e. decrease of IL-1β) and no adverse effects as observed for batyl alcohol (i.e. decrease of IL-10). In addition, DEA-CLA presented similar activity to CLA showing a trend to increase the secreted levels of adiponectin and decreasing the secreted levels of leptin.

**Conclusions:**

CLA and DEA-CLA modify adipocyte inflammatory mediators and also could play a role on energy homeostasis through depletion of leptin levels.

## Background

Obesity and its pathological complications, including atherosclerosis, hypertension, and insulin resistance, have increased to reach epidemic dimensions nowadays [1]. Some important factors for the development of these disorders are excessive accumulation of abdominal fat, which is known to play an important role in development of chronic inflammation; deposition of lipids into non-adipose tissues such as liver and muscles; atherosclerosis and chronic inflammation that increase risk in cardiovascular disorders and diabetes [2]. Adipose tissue is not just a site of energy storage but also behaves as a dynamic endocrine organ [3], it also plays an important role in energy expenditure, both as depot for energy-rich triglycerides as a source for metabolic hormones as well [4,5]. Adipocytes produce a large number of so-called adipokines, such as leptin, adiponectin, interleukin (IL)-1β, IL-6 and tumor necrosis factor-alpha (TNF-α). Some of these molecules affect energy metabolism and insulin sensitivity in other tissues such as muscle and liver [6]. During obesity, lipid storage in adipocytes is increased, which triggers the release of adipokines [7,8]. During inflammation, the mature adipocytes of the adipose tissue are responsible for increasing production of pro-inflammatory adipokines [9], including mentioned TNF-α, IL-1β, IL-6. That disregulation contributes to obesity and chronic inflammation [10]. The local increase of these adipokines have been directly related to insulin resistance, increasing lypolisis and increasing leptin levels [5]. Moreover, IL-6, as other proteins like TNF-α and C-reactive protein, is a clinic marker of cardiovascular risk [11].

Increased level of TNF-α in adipose tissue is closely associated with obesity-related complications such as insulin resistance [12], therefore providing useful therapeutic targets for modulating visceral obesity-related pathologies it is a must.

Besides secretion of pro-inflammatory adipokines, adipocytes are also responsible of leptin synthesis. Leptin is a hormone that plays an important role in the regulation of body mass index (BMI). In this case leptin acts regulating appetite and on the energetic expenditure as it encourages catabolic pathways versus anabolic pathways regulating 5'-AMP-activated protein kinasa (AMPK) in muscle and liver [2]. Furthermore, leptin can modulate the proliferation and differentiation of lymphoid cells of immune system and can induce the inflammatory response [5]. Also, leptin can lead pro-thrombotic states stimulating plaquetary aggregation at the same time that inhibiting the coagulation and fibrinolysis to show a pro-atherosclerotic effect [13].

Adiponectin is a hormone synthesized exclusively in mature adipocytes. Adiponectin is down-regulated in obesity, diabetes type 2 and in coronary diseases. It presents anti-inflammatory activity as inhibit the synthesis of TNF-α in adipocytes and in macrophages [13] through the modulation of NF-kβ [5]. Furthermore, anti-atherosclerotic effects of adiponectin has been described through its up-regulation in mouse model (ApoE -/-) of atherosclerosis in which the formation of atherosclerotic plaques is reduced [2].

An attractive way of reducing the chronic inflammatory process in obesity consists on the use of natural products, in particular, some compounds present in foods that have been using for a long time [14]. Some of these compounds, as alkoxyglycerols, have been described that decreases the levels of pro-inflammatory cytokines and leptin simultaneously and increase the levels of adiponectin, in other words, produce anti-inflammatory effects [15,16]. Whereas CLA is considered effective against obesity [16], batyl alcohol, on contrary, has been described as an inflammatory agent [17].

The aim of this study is to describe the effects of diesterefied alkoxylglycerols (DEA-CLA), products of esterification of CLA and batyl alcohol, with the activity of parental compounds and check if they present beneficial properties in an *in vitro *model of inflammation and other chronic diseases related to the inflammatory process using human mature adipocytes activated with TNF-α [18].

## Results

**Figure 1:**
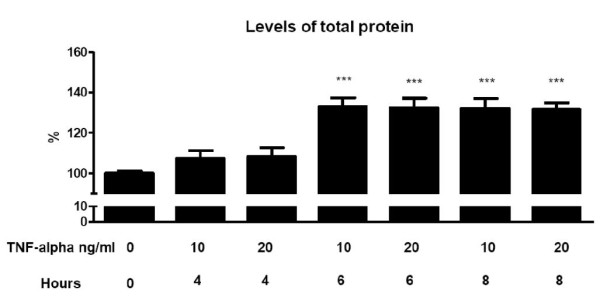
**Levels of total protein in TNF-α induced secretion in human adipocytes**. Results are shown as the means ± SEM of triplicate determinations. Statistic Dunnett's multiple comparison test VS Ctrl - TNF-α signification is represented by * P values less than 0.05 significant ** P values less than 0.01 very significant and *** P values less than 0.001 extremely significant.

In order to examine the effects of TNF-α on the adipokine secretions, fully differentiated human adipocytes were treated for different times (4, 6 y 8 hours) with several concentration of recombinant human TNF-α [19]. Proteins secreted into the medium were measured by the Bradford assay as mentioned in materials and methods. These TNF-α treated cells showed an increased in total protein secreted (Figure 1). The increase in protein secretion was used as indicator for adipocyte activation. The secretion of proteins increased from 4 hours to 8 hours, and the levels were stable at 6 hours at concentration of 10 ng/ml.

The composition of DEA-CLA analyzed by HPLC-ELSD can be seen in Table 1.

**Table 1:** DEA-CLA composition determined by HPLC coupled with Evaporative Light Scattering Detector, results expressed as Weight %.

DEA-CLA lipid composition	Weight %
diesterified alkylglycerols (DEAG)	78,4

Scualene (SQ)	8,8

CLA	10,1

Cholesteryl esters	2,7

**Figure 2:**
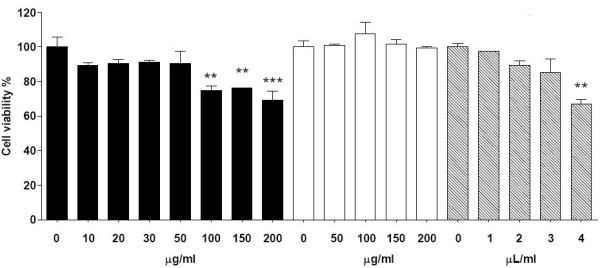
**Effects of alcoxyglycerols on mature adipocytes viability**. Cells were treated with increasing concentrations of batyl alcohol (from 0 to 200 μg/ml) (black bars), DEA-CLA (from 0 to 200 μg/ml) (white bars) and with (striped bars) CLA (from 0 to 4 μl/ml), for 24 h. Cell viability was determined by the MTT assay. Values represent the mean ± SEM of three independent experiments and statistic signification is represented by ** P values less than 0.01 very significant and *** P values less than 0.001 extremely significant.

Prior to examine the effects of lipids on adipokine secretions, it was studied the cytotoxicity of lipids candidates to study, batyl alcohol, CLA y DEA-CLA. So adipocytes were incubated with several alkoxyglycerols concentrations during 24 hours. Figure 2 shows the effects of the studied lipids in mature adipocytes. No significant decrease in cell viability was observed for concentrations lower than 50 μg/ml of batyl alcohol for incubations of 24 hours. Reduction in cell viability in a dose dependent manner was observed for higher concentrations than 50 μg/ml of batyl alcohol. Similar behavior was observed for CLA for concentrations higher than 3 μl/ml. DEA-CLA did not present cytotoxicity for concentrations lower than 200 μg/ml.

To investigate whether studied alkoxyglycerols could modify the TNF-α-induced secretion of adipokines by adipocytes, human adipocytes were pre-treated with TNF-α using 10 ng/ml for 6 hours. After this period, the induced adipocytes were treated with the highest not toxic concentration of each lipid (batyl alcohol 50 μg/ml, CLA 3 μl/ml and DEA-CLA 200 μg/ml) for 24 hours.

**Figure 3:**
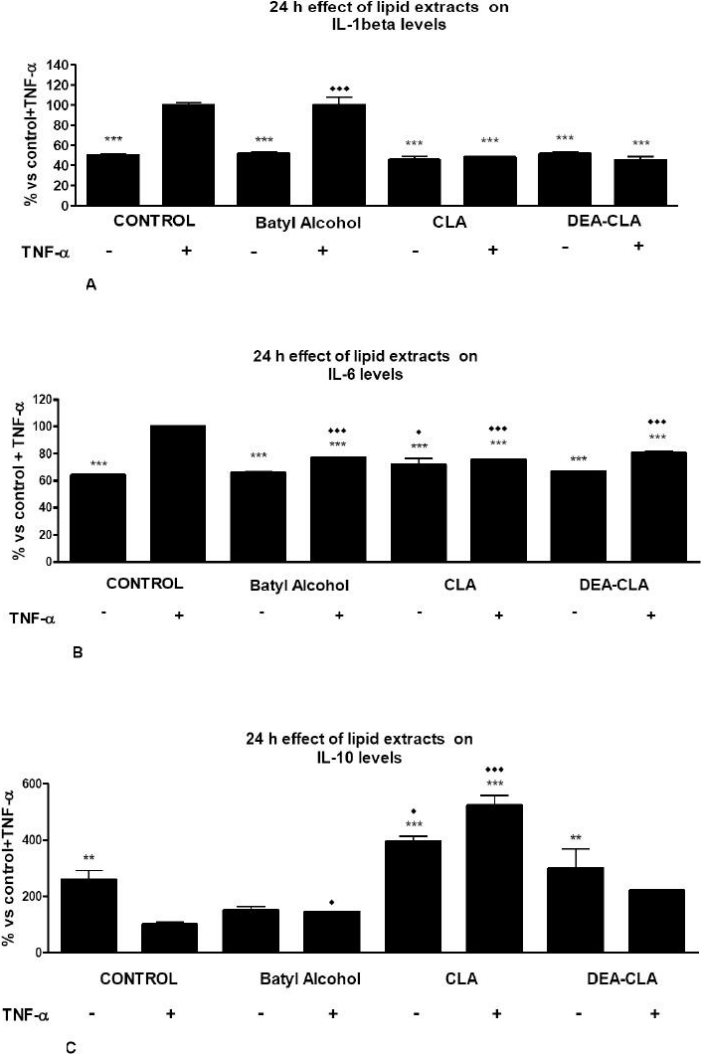
**Effects of alcoxyglycerol treatments on TNF-α induced adipokine secretion by human adipocytes**. After pre-treatment with 10 ng/ml TNF-α for 6 hours, human adipocytes were treated with 200 μg/ml DEA-CLA, 50 μg/ml batyl alcohol or 3 μl/ml of CLA. The secreted cytokines IL-1β (A), IL-6 (B) and IL-10 (C) into the medium were measured. Results are shown as the means ± SEM of triplicate determinations. Statistic Dunnett's multiple comparison test VS Ctrl +TNF-α signification is represented by * P values less than 0.05 significant ** P values less than 0.01 very significant and *** P values less than 0.001 extremely significant. Statistic "Bonferroni multiple comparison test VS Ctrl-TNF-α signification is represented by ◆P values less than 0.05 significant, ◆◆P values less than 0.01 very significant and ◆◆◆P values less than 0.001 extremely significant.

After 24 hours of incubation with CLA or DEA-CLA on TNF-α-induced cells, the pro-inflammatory adipokines IL-1β and IL-6 secretions decrease to reach the non TNF stimulates level of the control group. In other words, activation by TNF-α was inhibited and levels reached control levels (Figure 3A and 3B). Treatment with batyl alcohol did not affect secretions of non stimulated groups, but levels on TNF-α-induced group reached to non stimulated.

Regarding to the anti-inflammatory adipokine, IL-10, batyl alcohol decreased its level in both groups, TNF-α stimulated and non-stimulates. CLA increased significantly the secretion levels of IL-10, also in both groups. DEA-CLA showed to have lower effects than CLA but higher than batyl alcohol (Figure 3C).

**Figure 4:**
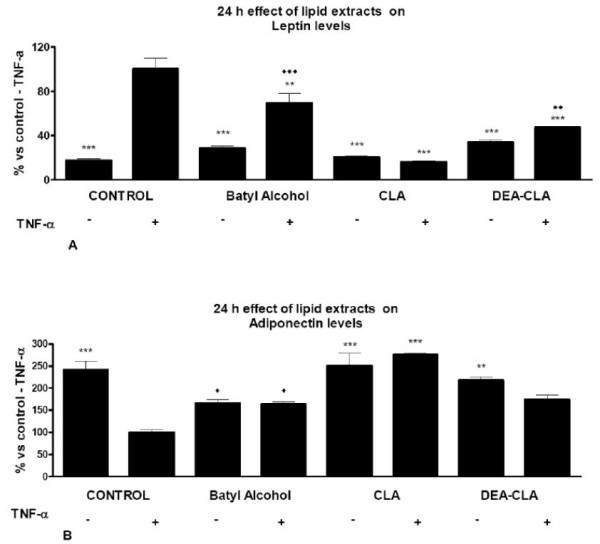
**Effects of alcoxyglycerol treatments on TNF-α induced adipokine secretion by human adipocytes**. The secreted adipokines leptin (A), adiponectin (B) into the medium were measured. Results are shown as the means ± SEM of triplicate determinations. Statistic Dunnett's multiple comparison test VS Ctrl +TNF-α signification is represented by * P values less than 0.05 significant ** P values less than 0.01 very significant and *** P values less than 0.001 extremely significant. Statistic "Bonferroni multiple comparison test VS Ctrl - TNF-α signification is represented by ◆P values less than 0.05 significant, ◆◆P values less than 0.01 very significant and ◆◆◆P values less than 0.001 extremely significant.

All lipid treatments reduced the secretion levels of leptin significantly in TNF-α-induced and non-induced cells. CLA < DEA-CLA < batyl alcohol recovered the secretion levels of non-TNF-α-induced control cells (Figure 4A).

On contrary, all treatments increased significantly the secretion levels of adiponectin in TNF-α-induced and non-induced adipocytes respect control levels. CLA < DEA-CLA < batyl alcohol recovered the secretion levels of non-stimulated control cells (Figure 4A). On contrary batyl alcohol decreased adiponectin levels in non-stimulated cells.

**Figure 5:**
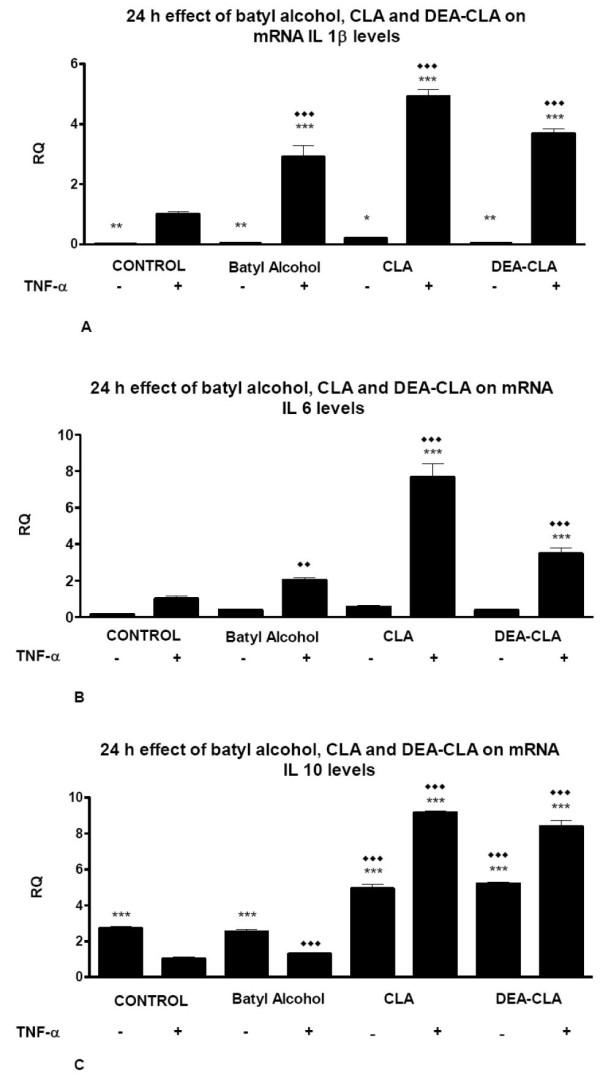
**Effects of alcoxyglycerols on the relative 24 h transcription gene quantification (RQ) of IL-1β (A), IL-6 (B) an IL-10 (C) on human adipocytes**. Data represent means ± SEM calculated from six independent experiments with 3 replicates for each treatment. Statistic Dunnett's multiple comparison test VS Ctrl +TNF-α signification is represented by: * P values less than 0.05 (significant), ** P values less than 0.01 (very significant), and *** P values less than 0.001 (extremely significant). Statistic Bonferroni multiple comparison test VS Ctrl - TNF-α signification is represented by: ◆◆ P values less than 0.01 (very significant), and ◆◆◆ P values less than 0.001 (extremely significant).

24 hours treatments of cells using any of the studies alkoxyglycerols caused a significant increment of IL-1β gene expression of stimulated cells in TNF-α-induced cells, but did not affect the gene expression of the non-stimulated groups (Figure 5A).

Similar results were observed for IL-6 in all treatments: gene expression increased in TNF-α-induced cells, but was not modify in non stimulated groups (Figure 5B).

IL-10 gene expressions were increased in cells treated with either CLA or DEA-CLA during 24 hours and higher increases were observed when cell were pre-stimulated by TNF-α. There were not any effects when cells were treated with batyl alcohol; levels remain in similar levels than their respective controls.

**Figure 6:**
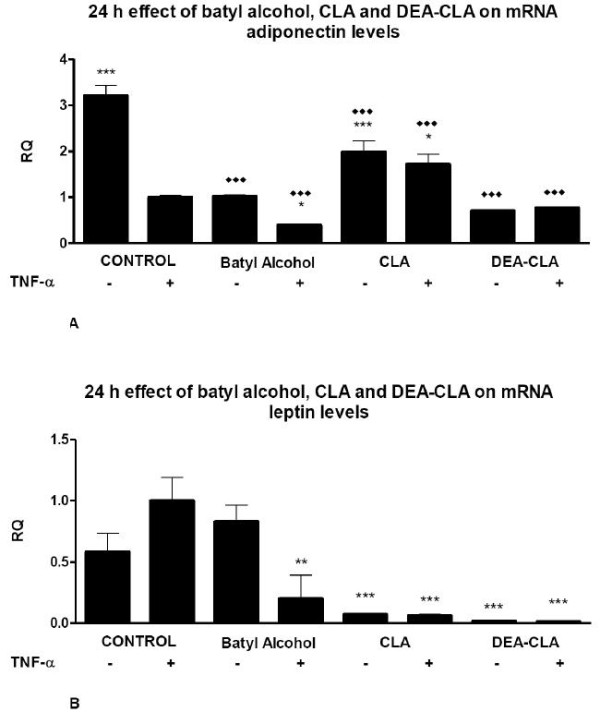
**Effects of alcoxyglycerols on the relative 24 h transcription gene quantification (RQ) of leptin (A) and adiponectin (B) on human adipocytes**. Data represent means ± SEM calculated from six independent experiments with 3 replicates for each treatment. Statistic Dunnett's multiple comparison test VS Ctrl +TNF-α signification is represented by: * P values less than 0.05 (significant), ** P values less than 0.01 (very significant), and *** P values less than 0.001 (extremely significant). Statistic Bonferroni multiple comparison test VS Ctrl - TNF-α signification is represented by: ◆◆ P values less than 0.01 (very significant), and ◆◆◆ P values less than 0.001 (extremely significant).

Adiponectin gene expression was, in general term, reduced in TNF-α-induced cells in all treatments (Figure 6A), even in non-TNF-α-induced cells although the reduction were less noticed in CLA treated adipocytes. Treatments with any lipids caused a significant reduction in adiponectin gene transcription.

All treatments decreased significantly the secretion levels of leptin in all groups, TNF-α-induced and non-induced adipocytes, respect control levels, except for batyl alcohol in stimulated cell groups (Figure 6B).

**Figure 7:**
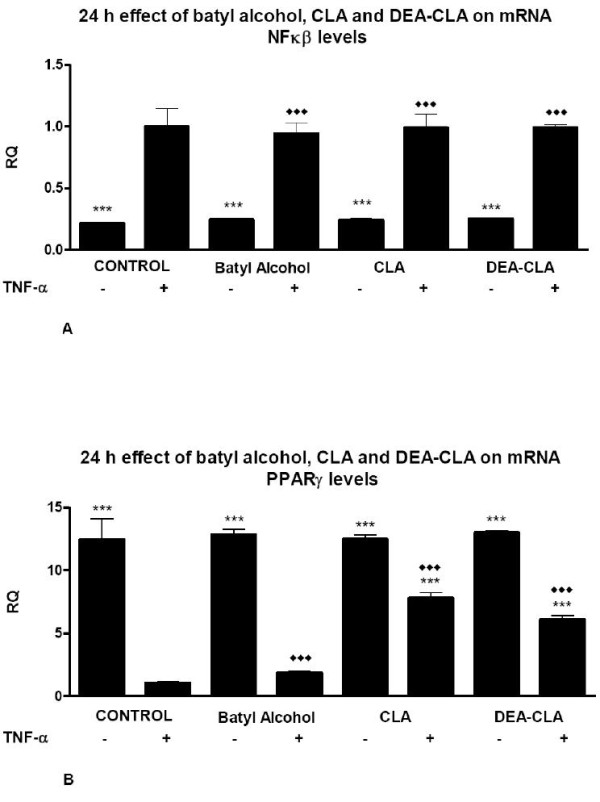
**Effects of alcoyglycerols on the relative 24 h transcription gene quantification (RQ) of NFκβ (A) and PPARγ (B) on human adipocytes**. Data represent means ± SEM calculated from six independent experiments with 3 replicates for each treatment. Statistic Dunnett's multiple comparison test VS Ctrl +TNF-α signification is represented by: * P values less than 0.05 (significant), ** P values less than 0.01 (very significant), and *** P values less than 0.001 (extremely significant). Statistic Bonferroni multiple comparison test VS Ctrl - TNF-α signification is represented by: ◆◆ P values less than 0.01 (very significant), and ◆◆◆ P values less than 0.001 (extremely significant).

Regarding to NF-kβ, none of the treatments with any of the studied alkoxyglycerols modified NF-kβ gene expression (Figure 7A). Levels observed in all treatments were similar to observed to TNF-α stimulated and non-stimulated control groups.

PPARγ gene expression were no modified in non-induced adipocytes group respect non-induced control but were increased in TNF-α stimulated groups when were treated either CLA or DEA-CLA although did not recover expression levels of non-induced control group (Figure 7B).

## Discussion

As demonstrated by Ouchi et al., the mature adipocytes are responsible of increased production of pro-inflammatory adipokines during inflammation [20]. Macrophage infiltration has been postulated to be a primary stimulus for the inflammatory properties of adipose tissue secreting high levels of TNF-α, which down-regulated the expression of PPARγ through the activation of NF-kβ [6,18].

Several natural compounds are known for their beneficial properties to some diseases or their derived complications and particularly concerning to their anti-inflammatory effects [18,19,21]. Some of these effects include inhibition of the TNF-α activated-NF-kβ signaling in adipocytes [18]. In the present study, it was studied if alkoxiglycerols could play a role modifying TNF-α-induced secretion of inflammatory adipokines in mature adipocyte cells.

Regarding to the non-esterified alkoxyglycerol studied, 3-octadecylglycerol (batyl alcohol), it was described in 1998 by Yamamoto et al. as a potent activator of the inflammatory process in mouse macrophages [17], but no extensive literature could be found for this lipid. In our experiments, batyl alcohol did not present a high pro-inflammatory trend but levels of anti-inflammatory adipokines, IL-10 and adiponectin were significantly reduced.

CLA and DEA-CLA presented anti-inflammatory activity in our experiments, since they decrease the secretion of IL-1β and IL-6, but not their gene expressions, and increase the IL-10 levels and gene expression. At the same time recovered the levels and expression of adiponectin and decreased levels of leptin secretions. CLA is commonly used as nutraceutical or as a functional food ingredient due to its anti-obesity effects, demonstrated in several studies [22,23]. DEA-CLA presented characteristic effects closer to CLA, than to batyl alcohol. Our data suggest that the esterification between the CLA and batyl alcohol produced a compound with properties with more benefits (i.e. decrease of IL-1β by CLA in stimulated cells) than drawbacks (decrease of IL-10 by batyl alcohol). Although, opposing results were obtained regarding the decreasing levels of IL-6 and IL-1β secretion and the increasing levels of their mRNA by CLA or DEA-CLA treatments in activated cells, this fact was previously described for CLA, which increased IL-6 and IL-1β gene expression in TNF-α stimulated adipocytes [21]. On the other hand, DEA-CLA also presented similar CLA activity since showed a trend to increase the secreted levels of adiponectin and to decrease secreted levels of leptin. All these changes could be mediated by PPARγ nuclear receptor, on TNF-α stimulated cells, since its gene expression strongly decreased after 24 h of incubation with any of the studied alkoxyglycerols but not in non-stimulated adipocytes. The mechanism of action could be independent from NF-kβ at 24 hours since its expressions was not modified on any treatment, although in general the inflammatory mediators linked to chronic inflammation have been shown to be regulated by NF-kB [24]. CLA consist on dienoic isomers of linoleic acid, including trans-10, cis-12 CLA and cis-9 trans-11 CLA. CLA isomers have been reported to reduced body weight, reduced leptin levels, increase PPAR γ and adiponectin, but the results are inconsistent because its been attributed to reverse causes at the same time, insulin resistance and inflammation [25,26]. Trans-10 cis-12 CLA but not cis-9 trans-11 CLA has been reported to antagonizes activity of PPARγ and consequently cells rescue mRNA levels of PPARγ [25]. On contrary, it has been demonstrated a reduced expression of PPARγ by TNF-α in adipocytes [27]. Consequently, controversial results have been reported about PPARγ expression [15,28] after trans-10 cis-12 CLA treatments: such as reduction of its gene expression in 3T3-L1 adipocytes [29] and in the other hand an increment of PPARγ expression in human adipocytes due a promotion of NFκβ activation followed by the subsequent induction of IL-6 [30].

For a deeper study of these alkoxygycerols on TNF-α stimulated adipocytes as cellular model of inflammation, more experiments should be carried out such as determinate secretion levels of other adipokines and their mRNA expression, ERK phosphorylation, COX-2 expression and quantification of its products PGE2, iNOS activity and expression.

## Conclusions

Our data suggest that DEA-CLA (product of the esterification between the CLA and batyl alcohol) presented characteristics effects closer to CLA than to batyl alcohol. In addition, CLA and DEA-CLA modify adipocyte inflammatory mediators and also could have a play on the energy homeostasis through depletion of leptin levels.

## Methods

### Reagents

1-O-Octadecyl-rac-glycerol or Batyl alcohol, with purity higher than 99% w/w, was purchased from Nikko Chemicals Co., Ltd. (Tokyo, JAPAN). CLA (purity of 90% w/w) was a gift of Natural ASA (Sandvika, Norway) according to ASA specification, CLA composition by Gas Chromatography and expressed as Relative area % were Palmitic acid C16: 0, 3.9%; Stearic acid C18:0, 2.3%; Oleic acid C18:1 C9, 10.3%; Linoleic acid C18:2 C9, C12, 1.6%; CLA C18: 2, conjugated, 81.8% (CLA Cis9, Trans 11 isomer, 36.7% CLA Trans10, Cis12 isomer 38.3%) TNF-α was purchase from R&D Systems. Preadipocyte Basal Medium, Fetal Bovine Serum (FBS), L-Glutamine, Penicilin, Streptomycin, Preadipocyte Differentiation Medium, Insulin, Dexamethasone, Indomethacin, 3-isobutyl-1-methylxanthine and DMEM/Ham's F-12 1:1 were purchase from Lonza, USA.

### Transesterification reaction

The enzymatic synthesis of diesterified alkoxyglycerols with CLA has been previously reported [31]. Briefly, batyl alcohol (500 mg) and CLA (818 mg) were added to a 30 ml flask and mixed by swirling. Then the lipase (10% w/w) was added. The flasks were placed in an orbital shaker (200 rpm) at 55°C. Samples (30 μL) were withdrawn periodically.

### Analyses of DEA-CLA composition

The analyses were effected on a kromasil silica 60 column (250 mm by 4.6 mm, Analisis Vinicos, Tomelloso, Spain) coupled to a CTO 10A VP 2 oven, a LC-10AD VP pump, a gradient module FCV-10AL VP, a DGU-14A degasser, and a evaporative light scattering detector ELSD-LT from Shimadzu (IZASA, Spain). The ELSD conditions were 2.2 bars, 35°C, and gain 3. The flow rate was 2 ml/min. A splitter valve was used after the column and only 50% of the mobile phase was directed through the detector. The column temperature was maintained at 35°C. The utilized mobile phase has been previously reported by our group [32].

### Cell culture

Human preadipocytes (Lonza, USA) were incubated in Preadipocyte Basal Medium containing 10% FBS, 2 mM L-Glutamine, 100 units/ml penicilin and 100 μg/ml streptomycin, at 37°C, 5% CO_2_, in a humidified incubator up to reach 85-90% of confluence. Cells were induced to differentiation into adipocytes by incubation with Preadipocyte Differentiation Medium containing insulin, dexamethasone, indomethacin and 3-isobutyl-1-methylxanthine for 3 days. After this time, cells were adhered to the culture dish and the medium was replaced every 3 days for 15 days. At the end of 15 days, cells were differentiated into adipocytes and lipid droplets into the cells could be visually appreciated. Afterwards, the Adipocyte Differentiation Medium was removed and the cells were starved in DMEM/Ham's F-12 1:1 for 1 day prior to assay with the plant extracts. Cells were activated with TNF-α (10 ng/ml) for 6 h and then treated with the different studied lipids for 24 hours. The supernatant of the different cultures were collected and analyzed for secreted adipokines (IL-1β, IL-6, and IL-10, leptin and adiponectin).

### Citotoxicity assay

Lipids toxicity was assessed using the mitochondrial-respiration-dependent 3-(4,5-dimethylthiazol-2-yl)-2,5-diphenyltetrazolium (MTT) reduction method. Preadipocytes cells were plated in 96 wells plates, differentiated and incubated with different concentrations of lipids for 24 hours at 37°C in 5% CO_2_. After treatment, the cells were washed with PBS and incubated with MTT 1 mg/ml in PBS for 2 hours at 37°C in 5% CO_2_. Afterwards, formazan crystals produced from MTT by the mitochondrial hydrolase, only activates in viable cells, were solubilized in lysis buffer (10% SDS in 50% dimetilformamide pH = 7) and the absorbance of each well was then read at 540 nm using a microplate reader (Sunrise Remote, Tecan). The optical density of formazan formed in control cells (without treatment with extract) was taken as 100% of viability.

### Bioactivity assay

Lipid extracts were dissolved in dimethyl sulfoxide (DMSO; Sigma-Aldrich) to stock concentration of 10 mg/ml determined as the maximum dose not toxic to cells in the citotoxicity assays. Mature adipocyte cells were platted and differentiated in 24 well plates. After differentiation, the cells were washed with PBS and activated with TNF-α (10 ng/ml) for 6 h and incubated with the lipids diluted in PBS free medium, for 24 at 37°C in 5% CO_2_. Afterwards, the supernatant was frozen and the cells RNA was isolated. Aliquots were analyzed to determine secreted cytokines, leptin and adiponectin.

### Total protein quantification

The Bradford method was used to determine the total protein content in the supernatant. 5 μl of supernatant was incubated with 250 μl of Bradford reagent (Sigma) for 30 min in the dark at room temperature. The absorbance at 595 was measured and the protein concentration determined using a standard curve.

### Enzyme-Linked Immuno Sorbent Assay for quantification of cytokines

The supernatant of the different treatment were collected. The concentrations of IL-1β, IL-6 and IL-10 were assayed using ELISA kits from BD Biosciences, and leptin and adiponectin were assayed using ELISA kits from R&D Systems. Absorbances were read at 450 nm with λ correction at 570 nm using a microplate reader (Sunrise Remote, Tecan Austria GmbH, Grödig, Austria). Each concentration was determined from the standard curve and expressed as % of non-activated controls.

### Total RNA isolation

Total RNA from adipocytes was isolated using the Trizol^® ^reagent from Invitrogen. 9.000 cells were homogenized in 200 μl of Trizol^® ^reagent and, if necessary, stored at -80°C. Following homogenization, samples were let to rest at room temperature for 5 minutes. Afterwards, 40 μl of chloroform was added, the tubes vigorously shaken for 15 seconds and let to rest at room temperature for 5 minutes. Tubes were then centrifuged at 12000 g (VWR, Galaxy 4D, diameter 14 cm), 4°C for 15 minutes. The aqueous (upper and colorless) phase was transferred to a new tube. 100 μl of isopropyl alcohol was added to the aqueous phase; the tube was then gently mixed and incubated at room temperature for 10 minutes. After incubation, samples were centrifuged at 12000 g, 4°C for 10 minutes. A gel-like pellet was formed and the isopropyl alcohol removed. The pellet was washed with 200 ml of 75% Ethanol in DEPC treated H_2_O, and centrifuged at 7600 g, 4°C for 5 min. The ethanol was then removed and the pellet let to dry until colorless. Total RNA was then dissolved in 15 μl of DEPC H_2_O, incubated at 55°C for 10 minutes and stored at -80°C for future use.

### Gene expression quantification

IL-1β, IL-6, IL-10, leptin, adiponectin and 18sRNA gene expression were quantified using real-time PCR. 10 ng/μl of total RNA isolated from mature adipocytes cells was used as template for cDNA synthesis using the High Capacity Archive Kit from Applied Biosystems, according to the manufacturer's instructions. Real-time PCR was performed using Taqman Probes (Applied Biosystems) following the manufacturer's recommendations. The Taqman probes used were: Hs99999029_m1 for IL-1β, Hs00174131_m1 for IL-6, Hs999999035_m1 for IL-10, Hs00174877_m1 for leptin, Hs00605917_m1 for adiponectin, Hs00765730_m1 for NFκB, Hs01115729_m1 for PPARgamma and Hs99999901_s1 for 18sRNA. Gene expression levels were then normalized to 18sRNA expression and compared to it.

### Statistical analysis

All data were expressed as the mean ± SEM. For single variable comparisons, Student's t-test was used. For multiple variable comparisons, data were analyzed by one-way analysis of variance (ANOVA) followed by Dunnett's test using SigmaStat statistical software (Windows Version 5.0 Systat Software Inc., Point Richmond, CA, USA). P values less than 0.05 were considered significant.

## Competing interests

The authors declare that they have no competing interests.

## Authors' contributions

AO conceived the study, its design and coordination, performed the statistical analysis and drafted the manuscript. CGA participated in all the bioactivity assays (citotoxicity, ELISA and gene expression quantification). EAG participated in all the bioactivity assays (citotoxicity, ELISA and gene expression quantification). CT carried out the trans-esterification reaction and the analyses of compositions. FJS participated in the design of the study. GR participated in the design of the study. All authors read and approved the final manuscript.
